# A Manual of Ascultation and Percussion

**Published:** 1866-12

**Authors:** 


A Manual of Auscultation and Percussion. By M. Barth and M. Henri Roger. Translated from the sixth French edition. Philadelphia: Lindsay & Blakiston. 1866.

This is an abridgement of the larger work by the same authors. It is a small volume of 161 pages, very neatly printed and bound. Its style is simple and quite free from technicalities, and its arrangement of topics good. It is an excellent manual, both for students and practitioners.

For sale by S. C. Griggs & Co., Lake St., Chicago.

